# Nurse educators’ experiences regarding management practices at a nursing education institution

**DOI:** 10.4102/hsag.v27i0.1935

**Published:** 2022-11-21

**Authors:** Gugu M. Ndawo

**Affiliations:** 1 Department of Nursing, Faculty of Health Sciences, University of Johannesburg, Johannesburg, South Africa

**Keywords:** assessment, leadership, learning, management practices, nursing education institution, self-leadership, teaching, transcendental

## Abstract

**Background:**

Currently nursing education is undergoing major transformation that pose considerable challenges with which nursing education institution (NEI) management must deal. Yet nurse educators displayed behaviour that reflected distrust, loss of respect and loyalty and a paucity of admiration towards NEI management.

**Aim:**

The article aimed at exploring the lived experiences of nurse educators regarding the management practices at a NEI.

**Setting:**

The setting was a public NEI in Gauteng, South Africa that had 11 departments and offered both undergraduate and post-basic studies.

**Methods:**

A qualitative, exploratory, descriptive, contextual and phenomenology study was conducted with a purposive sample of 20 qualified nurse educators who were fully employed, taught at the current NEI and were willing to participate. Data were collected from June 2015 to July 2016 through face-to-face, semi-structured individual interviews and analysed using Tesch’s protocol. Ethical principles were observed and trustworthiness ensured.

**Results:**

The themes that emerged were management’s dominant use of one leadership style with inconsistent treatment; lack of stimulation to aspire to higher academic levels; and lack of support with minimal resources.

**Conclusion:**

Nurse educators experienced ineffective management practices and this ineffectiveness had negative impact for nurse educators in coping with major transformational changes brought by the dynamic nursing education environment.

**Contribution:**

The recommendations made might assist NEI managers to improve their management practices, therefore assist nurse educators cope with transformation. The findings added to the body of existing knowledge on effective management of NEIs by aiming to achieve institutional and individual goals within a transformative environment.

## Introduction

Management practice refers to the methods and innovations that managers use to achieve intended organisational goals in a complex, challenging and ever-changing environment, and it includes training and empowering of staff as well as introduction of quality improvement plans and various forms of new technology (IGI Global 2022). Nursing education institutions (NEIs) are not exempted from this definition because if they are also properly managed – planned, organised, led and controlled – they can successfully achieve their organisational goals despite the dynamic external factors. However, NEI management practice is a complex, challenging process, an impetus without which quality nursing education may not occur and to which the success or failure of the NEI can be attributed (Alshammari 2018). Effective NEI management practice is extremely crucial currently as nursing education is undergoing a major transformation that poses considerable challenges and dilemmas (Matlakala 2017), which NEI management must contend with.

The current NEI is also undergoing a major transformation that includes the introduction of new nursing qualifications in accordance with the international standards wherein a bachelor’s degree (Regulation [R.]174 of 2013) is required for registration as a professional nurse and midwife (South African Nursing Council [SANC] 2013). Prior to this major transformation, the NEI offered a number of diploma programmes that included R.425 of 1985, which led to a professional nurse and midwife qualification (SANC 1985) that subsequently became a legacy qualification in 2018. However, for the NEI to be able to offer the new nursing qualifications on the third band of the National Qualification Framework, for example, the bachelor’s degree, it is expected to be accredited and registered with the Department of Higher Education and Training as a Higher Education Institution (HEI) (Armstrong, Geyer & Bell 2019; Department of Health [DoH] March 2013; SANC 2010). The new nursing qualifications also necessitate the development and accreditation of innovative curricula and new teaching, learning and assessment (TLA) strategies for the new programmes (Zwane & Mtshali 2019).

In addition, the transformation requires that nurse educators hold a master’s degree to be able to teach the bachelor’s or hold an academic qualification at least one level higher than the level of the programme in which they will teach (Bvumbwe & Mtshali 2018; SANC 2020). The requirement means that there is a further demand on NEI management to capacitate nurse educators to meet the higher education standards such as scholarship of teaching and learning, research, publications and community engagement (Direko & Davhana-Maselesele 2017). A requirement that pose as a challenge in the current NEI because of limited number of nurse educators who hold a master’s degree (Armstrong et al. 2019). Prior to this major transformation, the NEI was affiliated with a university-based nursing department, which ensured that the programmes offered at the NEI were of quality and standard and provided necessary guidance and academic support. However, the affiliation is no longer in force since the termination of the legacy qualifications, and the NEI is now expected to provide higher education programmes under the Ministry of Higher Education and Training (Matlakala 2017; Zwane & Mtshali 2019) and must, according to the *Higher Education Act (No. 101 of 1997)*, also be a juristic person, that is, a HEI (Armstrong et al. 2019).

The NEI managers as the main drivers and agents of change, growth and development among their followers are expected to use innovation, creativity and flexibility to ensure continuous delivery of quality TLA while creating an environment within which effective transformation is achieved (Dinh, Caliskan & Zhu 2020; Gupta & Gupta 2021). However, being an agent of change is challenging, intimidating and to some degree, terrifying (Haage et al. 2021) because NEI managers are expected to conduct their day-to-day management duties while carefully handling the transformation process and ensuring that it is a success. Juggling between these duties require that they utilise critical thinking in making critical decisions and apply strategic thinking skills to solve problems effectively and deal with unrelenting transformational changes (Kri, Scott & Scott 2021; Matlakala 2017; Zwane & Mtshali 2019). It is for this reason that NEI managers depend on an effective leadership style to achieve the organisational goals (Bakker et al. 2022).

### Theoretical framework

The transformational leadership (TFL) theory underpins this study, and it serves as the lens through which the problem statement is supported and data are analysed. Transformational leadership is a process where a leader works with their followers in advancing one another to higher levels of engagement, motivation and morality to achieve their potential (MacNeil, Dwyer & Matear 2021). The TFL theory posits that humans are a critical asset whom, when assisted and supported, become more committed to effectively achieve organisational goals (Ghasabeh & Provitera 2017). The goal of TFL is to transform followers as individuals by supporting them so that they yearn for change and improvement and, at the same time, embrace being led (Leppard 2021).

The authentic transformational leaders utilise the four ‘*I*’ s, viz, idealised influence, inspirational motivation, intellectual stimulation and individualised consideration to transform their followers and positively influence them to achieve organisational goals (Senekal & Lenz 2021). They influence their followers by broadening their understanding of the importance of the organisational task at hand while maximising their individual self-interest by incorporating followers’ own individual development needs for the success of the organisation (Bakker et al. 2022). Within the transformational environment, followers thrive and respond by feeling trust, admiration, loyalty and respect for their leader and therefore put more effort in working harder than they are originally expected. Subsequently, the follower’s self-concept is influenced as their self-efficacy, confidence and self-esteem are enhanced. The transformational relationship that is built exceeds the mundane activities of the organisation and assists both leaders and followers to achieve greater level of self-actualisation (Burns, cited in Bakker et al. 2022). However, importantly for any leader to note is that on the contrary, TFL gives pseudo-transformational leaders too much power ‘to determine both the ends and means of collective action, which can incentivise narcissism and hubris’, which are the antithesis of the TFL goal (Leppard 2021:47), the power that NEI managers should guard against.

The TFL theory is an appropriate framework for this study because it is a leadership style that is considered appropriate to motivate NEI managers to deal effectively with the contemporary major transformation challenges encountered by educational institutions (Nurtjahjani et al. 2021). It is also pertinent for use by NEI managers who seek to transform nurse educators by creating ideas, new perspectives and a new path towards their growth, development and sustainability in the complex, challenging and ever-changing nursing education environment (Jenkins 2021). The researcher is, therefore, of the opinion that NEI managers can certainly make a positive and lasting transformational change in the life of nurse educators, and when they use the four ‘*I*’ s, transformation in nurse educators would occur. Nurse educators would then respond positively to any NEI challenges brought forth by the dynamic nursing education environment.

### Problem statement

Twelve nurse educators were observed by the researcher to be not engaging in clinical accompaniment of students, however presented clinical registers to NEI management as if they were. They anecdotally based their misconduct on the perception of not being properly managed by NEI management. Similar findings were reported by Tshabalala (2017) where it was found that NEI’s management ineffectiveness subsequently led to nurse educators awarding high marks of up to 100% theoretically to students who later exhibited poor clinical performance. The nurse educators’ misconduct demonstrated the TFL characteristic trait of *idealised influence,* which is defined as the way in which the leader is perceived by their followers, the impact they have on their followers and the way they, in turn, affect their feelings and behaviours (Bakker et al. 2022). Nurse educators’ inappropriate behaviour reflected distrust, loss of respect and loyalty and a paucity of admiration towards NEI management, and it resulted in less devotion and willingness to pursue the goals of the organisation.

Gılıç and İnandi (2021) warned that once educators, as important stakeholders of educational institutions, develop negative perceptions such as inappropriate management and non-support from their managers, their enthusiasm for teaching decreases and they engage in defiant workplace behaviours. Furthermore, Bvumbwe and Mtshali (2018) found that ineffective management results in the paucity of needed competencies among graduates attributable to the inadequate productive capacity of NEIs. Overall, ineffective management activities place NEIs at risk of failing in their endeavour to empower nurse educators and produce skilled global professional nurses attributed to lack of accountability and responsibility by nurse educators (Nyoni & Botma 2020). Effective management practices are indispensable now more than ever in South Africa as NEIs transition to HEIs. The researcher was particularly unaware of any existing research that explored nurse educators’ experiences of management practices in the current NEI and therefore felt that there was a necessity to explore such experiences and the effect the management practices have on TLA through the research question: ‘What are the experiences of nurse educators regarding management practices at a nursing education institution?’ Understanding the lived experiences of the nurse educators will hopefully lead to recommendations needed to enhance good management practices.

### Aim and objectives

The purpose of this article was to explore and describe the lived experiences of nurse educators regarding management practices at a NEI with a view to present recommendations that can enhance good management practices at a NEI.

## Research methods and design

### Study design

A qualitative, exploratory, descriptive, contextual and phenomenology research design was employed to gain a comprehensive understanding of participants’ experiences with the purpose of making sense of them (Gray, Grove & Sutherland 2021). The researcher used this design to explore the complex experiences related to the phenomenon of interest and generate descriptions that were rich, thick and deep from the nurse educators’ lived experiences, thereby expanding the understanding of the management practices in the current NEI. The researcher situated herself into the world of the participants and therefore was enabled to visualise their world as they have experienced it, a possibility afforded by the qualitative research (Creswell & Poth 2018). The insiders’ perspectives of NEI’s management practices and their effect on TLA as interpreted by nurse educators were obtained. Through the use of this design, the profundity, richness and intricacies embedded in the lived experiences of nurse educators were pursued, explored and described in their natural setting.

### Setting

The research was contextual in nature because the natural setting was a public NEI in Gauteng, South Africa. The NEI offered a four-year comprehensive diploma programme leading to registration as a nurse (general, psychiatric and community) and midwife under R.425 of 1985, as amended (SANC 1985). Three post-basic qualifications in nursing, namely, community, midwifery and primary healthcare nursing diplomas were also offered by this NEI. There were 11 departments that included eight academic and three non-academic departments that were led by nine heads of departments. The eight academic departments were led by eight heads of departments and the remaining three non-academic departments were jointly led by one head of department.

### Research method

Phenomenology was used as a method to discover and subsequently describe in depth the essential, collective meaning of nurse educators’ lived experiences (Kim et al. 2020). The discovery and description of the lived experiences were facilitated and achieved through the use of intuition and bracketing. The researcher used intuition by remaining open to the meaning of the lived experiences of nurse educators. Therefore, the information was received as purely as possible without any interpretation and as communicated by nurse educators while concentrating on the management practices at the NEI as the phenomenon of interest. Furthermore, the researcher remained open to the meaning of the data by verifying the information with the participants as well as describing the phenomenon as shared by them. Bracketing was used so that the phenomenon would reveal itself by actively withholding and striping the researcher’s cognisance of own prior knowledge and experiences during data collection and analysis while highlighting the collective essences embedded in nurse educators’ lived experiences. Furthermore, a reflexive diary was kept by the researcher and was given to the independent coder to analyse, describe and interpret the researcher’s behaviour and responses, thereby pointing out biases on the analysed data (Qutoshi 2018).

### Sampling strategy

The accessible population comprised 104 nurse educators who qualified as such with the SANC. A purposive sampling method was used to consciously select nurse educators because the researcher believed that they would provide rich and comprehensive information on which a great deal about the management practices and their effects on TLA could be learnt. A total sample consisted of 20 nurse educators who willingly participated in the research.

The inclusion criteria comprised nurse educators who were registered with the SANC under R.118 of 1987 (SANC 1987). They taught students registered for either undergraduate or post-basic programmes, have been employed full-time for a minimum of two years at the NEI and were willing to participate in the research.

### Recruitment

The names and contact details of nurse educators were requested and obtained from the office of the NEI principal after obtaining permission to conduct the research. The researcher then contacted nurse educators and a simplified information letter that contained no research jargon was sent to them through electronic mails (emails). The information letter detailed the invitation and described the purpose of the research, its envisaged benefits and risks, how data were to be collected, the approximated duration of each interview as well as description of how anonymity and confidentiality would be ensured. Furthermore, the information letter informed the prospective participants that participation in the research was voluntary and that they could retract from participation at any time without any adverse consequences. Moreover, the information letter contained the contact details of the researcher and those of the chairperson of the University’s Research Ethics Committee. Therefore, the prospective participants were given an opportunity to seek clarity and ask questions about the research prior to making an informed decision about participating. As nurse educators called and responded back, the researcher then scheduled appointments with them and reminder messages were sent via email 1 week before the set date of the interview.

### Data collection

The researcher collected data from June 2015 to July 2016 using face-to-face phenomenological, semi-structured, individual interviews during practical blocks, which ensured that TLA activities were not disrupted. The individual interview method was chosen because it allowed flexibility, availability and accessibility of each nurse educator wherein the date, time and venue of their preference were honoured, thereby allowing them greatest opportunity to share their lived experiences freely (Gray et al. 2021). Fourteen interviews were conducted in the NEI offices, while the other six nurse educators were interviewed in the comfort of their own homes. The interviews were conducted in English language and lasted between 45 and 60 min each. The central question that was posed during each interview was: ‘What are your experiences of management practices in your nursing education institution?’ Follow-up questions, probing, listening, reflecting, paraphrasing and summarising were employed to ensure in-depth exploration of the lived experiences (Murphy & Dillon 2015). Data redundancy was reached with the 14th participant, and six participants were further interviewed to confirm it; therefore, data saturation was achieved with the 20th participant. An audio-recorder was used during the interviews to capture accurately the participants’ exact words for subsequent verbatim transcription through replays. The collected data were further enriched through taking field notes by the researcher using a small diary to document the events, first impressions, conversations, participants’ emotions and behaviours that were observed during data collection as well as the researcher’s reflections on them (Phillippi & Lauderdale 2018).

### Data analysis

The collected data were transcribed verbatim directly after each interview. The researcher manually analysed the data using Tesch’s open coding protocol of qualitative data analysis (Creswell & Poth 2018). This data analysis method was chosen because it entailed the analysis of qualitative data through eight iterative, descriptive and logical steps. Each transcript and its field notes were read repeatedly to immerse in data and figure out what the text was about to determine its underlying meaning. The thoughts and ideas that came to the researcher’s mind while reading each transcript were jotted down in the margins. A list of topics was developed after rereading and margining all the transcripts. Further analysis of the list resulted in clustering together similar topics that were later assigned codes, which were then written in the transcripts and were aligned with the appropriate segments of the text. After coding all 20 transcripts, most descriptive wordings for the topics were identified and were converted into categories. The researcher then decided on the code for each category and assigned a final code, which was used to identify and conclude on the themes and sub-themes. An initial analysis of each theme was performed, and the existing data were recoded when the need arose. A second analysis was performed by an independent coder with expertise in qualitative data analysis using Tesch’s method who independently analysed the raw data that included the verbatim transcripts, interview recordings, field notes and the researcher’s reflexive diary. A consensus discussion was later held between the independent coder and the researcher in which an agreement was reached on the independently identified themes (Creswell & Poth 2018). Follow-up interviews were organised with participants who were selected randomly to verify the accuracy of the transcripts and the identified themes that they were true accounts of what they had conveyed during data collection. The follow-up interviews proceeded until a repetitive pattern of verification of accuracy was affirmed at the 11th participant. That was the point when the verification process was stopped.

### Trustworthiness

The four strategies of qualitative trustworthiness, namely credibility, transferability, dependability and confirmability were employed (Lincoln & Guba 1985; Rose & Johnson 2020) and are presented in Table 1.

**TABLE 1:** Strategies of qualitative trustworthiness.

Strategy	Application
**Credibility**	
Prolonged engagement	The researcher presented herself to conduct the phenomenological, semi-structured, individual interviews and spent sufficient time of a year and 1 month with the participants during data collection. Time was also afforded to the participants for self-expression and asking of questions related to the research. Triangulation was ensured by taking field notes for observation of verbal and non-verbal communication and by using primary and secondary sources in conceptualising the findings.
Member checking	Eleven follow-up interviews were organised with participants who were selected unsystematically to verify the accuracy of their accounts and the themes.
Reflexivity	The independent coder was given the researcher’s reflexive diary, which was kept throughout the research process that had all field notes with intuitive notes included that assisted in bracketing. Two colleagues who were not part of the research assisted with peer reviews to ensure the value of truth, one colleague held a PhD and was an expert in qualitative research and the other was conducting her own PhD study.
Authority of the researcher	The researcher was pursuing a doctoral study and held a master’s degree in qualitative nursing research.
**Transferability**	
Thick description	A dense description of the research methodology, the research context and purposive sample was presented to enable other researchers interested in the replication of the research, to do so. The presented findings were supported with verbatim quotes from participants and field notes. A literature control was conducted to determine the findings’ applicability.
**Dependability**	
Dense description	All the audio-recorded raw material, transcripts and signed consent forms were kept under lock and key and were strong password protected for the purposes of conducting an inquiry audit. A code-recode procedure was employed during data analysis.
**Confirmability**	
Confirmability audit trail	Informed consent was obtained from the participants after they were afforded the time to review the information letter; their questions were answered truthfully and their freedom of expression was encouraged. An independent coder who held a PhD and was well versed in qualitative data analysis was used.

### Ethical considerations

Ethical principles were observed based on those of respect for persons, beneficence and non-maleficence and justice (Dhai & McQuoid-Mason 2020; DoH 2015). Ethical clearance was granted by the Faculty of Health Sciences’ Research Ethics Committee of a public university in Johannesburg (REC-01-123-2014), Gauteng, and permission to conduct the research was granted by the NEI management. The researcher was a lecturer at a public university in Johannesburg while appointed as an external moderator for a nursing science subject at the current NEI. Informed consent to participate voluntarily and to audio-record the interviews was obtained from each participant after explaining the purpose and objectives of the research study. The researcher informed the participants of their expected participation role in the research, and participants were allowed to ask questions, which were answered truthfully prior to commencing with the interview. Self-selected numbers from 1 to 20 were used as pseudonyms and for the identification of transcripts to ensure anonymity. All data were either kept under lock and key or in a computer encrypted with a strong password to ensure confidentiality. Only the researcher and the independent coder who signed a confidentiality agreement had access to the data (Dhai & McQuoid-Mason 2020; DoH 2015). Participants were advised of their right to retract from participation at any time without any adverse consequences. Participation posed no risks or harm to participants, and no inducements were provided for it. No nurse educator was excluded during the recruitment process, none withdrew from research and only those who voluntarily consented were included until data saturation was achieved.

## Results

The results are presented in Table 2 and entail the demographic information of the participants as well the themes that emerged from the collected data.

**TABLE 2:** Demographic characteristics of participants.

Sample	Gender	Age (years)	Ethnic group	Teaching experience (years)	Higher qualification studies
*N* = 104; *n* = 20	Females = 17; Males = 3	37–65	Black people = 16; white people = 2; Indian = 1; mixed race = 1	3–12	Master’s = 15; Doctoral = 5

A summary of the demographic characteristics of the participants is presented in Table 2.

### Themes

Three themes that emerged from data analysis were management’s dominant use of one leadership style with inconsistent treatment, lack of stimulation to aspire to higher academic levels and lack of support with minimal resources. Each theme is elaborated on and supported with verbatim quotes that are presented in italic and the field notes in bold.

#### Theme 1: Management’s dominant use of one leadership style with inconsistent treatment

Nineteen participants experienced that NEI management dominantly used one leadership style where some nurse educators were left to do what they wanted whenever they wanted. They were also treated differently to some extent, with some nurse educators getting away with doing what and how they pleased, while others were regulated. Such leadership style left them unable to evaluate what was crucial from that which was trivial to TLA. Some participants said:

‘… [*F*]or the fact that we do what we like, it says a lot about our leaders … you make up your own mind.’ [*Sounding sarcastic*] (Participant 19, doctoral study, 57 years old)

‘… [*W*]e are not treated the same with others doing the “laissez-faire” and others “iron fist” [*holding out a fist*] … quite painful … you fail to concentrate on your teaching, learning and assessment work.’ [*Tearing up*] (Participant 4, master’s study, 43 years old)

Moreover, the treatment from NEI management was inconsistent, unequal and unfair, which created an unhealthy work environment for them and negatively affected their TLA activities. One participant stated:

‘… [*M*]y expectation is that management treats us equally and fairly … it’s not like that … here, the treatment is not the same … this is unhealthy and unfortunate for me and my job ….’ [*Seemingly unhappy*] (Participant 2, master’s study, 37 years old)

#### Theme 2: Lack of opportunities for postgraduate studies

The participants experienced a lack of opportunities for them to further their studies and support that made them feel they could not succeed in their own academic endeavours, including those obligated by new nursing educational changes. Some nurse educators remained in nursing education because of the favourable working hours, others had no desire to pursue higher degree studies, while those who were studying confirmed a lack of management support. These experiences were supported by the following quotes:

‘Now we are expected to be a higher education institution and we must all have masters and doctorates … I don’t think I can succeed with my doctorate, teaching and other roles with no support from management.’ [*Sounding frustrated*] (Participant 3, doctoral study, 40 years old)

‘… I don’t even see the need to have any higher degree as I have no inspiration, energy and certainly no support from above. These higher studies will leave me with no time for TLA … I’m mere staying at the college for the [*working*] hours ….’ (Participant 12, master’s study, 47 years old)

On the contrary, one nurse educator experienced that when she took the lead and told the NEI managers what their obligations were, they had no choice but to oblige. The participant said:

‘I … [*pointing at herself and sounding serious*], tell them what is expected of them in terms of my studies, for if you don’t, they just don’t listen or hear you [*placing emphasis*]. You must just take the lead … I do ….’ (Participant 5, master’s study, 40 years old)

#### Theme 3: Lack of support with minimal resources

The findings of this research also revealed that participants worked with minimal resources that worsened their workload and negatively affected their TLA activities. Resources included human, material and technological. The following quotes expressed this experience:

‘… [*T*]here are certain administration duties that are a must for me but there are also those that are a waste of my teaching, learning and assessment time … management does nothing about that.’ [*Sounding irritated*] (Participant 17, doctoral studies, 63 years old)

‘Management employed only one IT specialist … who must serve a total of about a hundred lecturers … should something go wrong with the computers in class … who will be available to assist you within reasonable time … this means taking away time allocated for facilitating ….’ (Participant 15, master’s study, 40 years old)

‘I’m sitting here in my office, I have a shared computer but I don’t have access to internet … these students have gadgets to search current information. Then here I come with the information that was written 10 years back … it affects the very TLA.’ (Participant 7, master’s study, 41 years old)

## Discussion

The research aimed at exploring the lived experiences of nurse educators regarding the management practices and their effect on TLA at a NEI. The research drew attention to several critical issues that were of great concern, which are conceptualised next.

### Theme 1: Management’s dominant use of a leadership style with inconsistent treatment

The leadership style that NEI management used was experienced by nurse educators as that of predominantly being absent, which left them doing what they desired without consideration of the consequences for the students. The absence of managers sometimes left nurse educators destitute with no support, guidance, constructive feedback or encouragement. The absence may unintentionally leave nurse educators unchecked with enormous autonomy and independence that permit vulnerability and leave nurse educators at risk of failing in their TLA activities, leaving NEI’s goals unachieved (Mabasa 2018). According to Dinh et al. (2020), nurse educators need guidance at all times, and therefore, NEI managers need to be actively present, involved, proactive, assertive and at times make undesirable, complicated decisions to guide them into exceptional practices for TLA activities to develop quality, globally competitive 21st-century graduates. The absence of NEI managers denotes a laissez-faire leadership style.

A laissez-faire leadership style is a passive-avoidant leadership, characterised by the absence of action in addressing critical institutional situations (Gilbert & Kelloway 2018). When such leadership is used, nurse educators participate less and do not engage in their work. There is lurking ambiguity about their responsibilities and duties, which results in evasion and abandonment of their responsibilities and a low sense of accomplishment evident in their irresponsible behaviour. Those treated with an ‘iron fist’ feel that management had ‘their favourites’ that has negative effects for the ‘non-favoured’, including resentment, which negatively affect their TLA performance (Webster, Brough & Daly 2016). Treatment of this nature is usually viewed as harmful, toxic and destructive (Baloyi 2020). On the opposite end of this leadership, authentic transformational leaders are proactive and always engaging with their followers and are successful in attaining organisational and followers’ goals because of their constant assessment of where the followers are and ensuring that their needs are satisfied and valued. Therefore, they never leave them behind or unclear of what is to be accomplished (Leppard 2021). On the contrary although, Miller (2018) argued that laissez-faire leadership style is effective when employed on individuals who form a strong team of highly skilled, motivated, competent and committed individuals with high levels of autonomy who make their own well informed decisions and equally achieve the desired organisational outcomes.

The participants also experienced inconsistent treatment from NEI managers. According to Dinh et al. (2020), NEI managers should, always, model the personal core values of effective leaders, such as fairness, equality and emotional intelligence, because feelings of inequality and unfairness signify definite disparities in leadership treatment in the workplace. The inconsistent and unfair treatment created unwanted, undesirable and unnecessary feeling that some nurse educators were better and more important than others. It inherently created an unhealthy environment that was stressful and led to feelings of dissatisfaction, resulting in a lack of commitment towards the NEI and its TLA activities. Communities of practice that are supposed to collaboratively and collectively practise excellence in TLA are not fostered. Moreover, such environment bred and enabled unnecessary academic silos, mistrust, professional jealousy and division among nurse educators, resulting in underperformance, low-quality work and underachievement of students’ and nurse educators’ own academic goals (Baloyi 2020).

### Theme 2: Lack of opportunities for postgraduate studies

The feelings of being unable to succeed were prominent in the face of other more advanced academic roles, such as being a teacher, facilitator, researcher, service provider and conducting community engagement, while having family responsibilities, all to be performed without management support. The inability to balance all the expected roles resulted in role interference, which occurred when a nurse educator with numerous roles had to undertake them simultaneously (Cabatan, Grajo & Sana 2019). Nurse educators are sometimes compelled to take their work home or work overtime to complete the designated workload.

The participants also shared that they were staying at their job because of the beneficial working hours. According to Mabasa (2018), continuance commitment in the workplace is a desire to stay because of the benefits the job offers. However, extreme levels of this commitment result in emotional exhaustion, declining feelings of accomplishment and organisational citizenship, which lead to inadequate performance (Ogunsola, Fontaine & Jan 2020). Nurse educators could be inspired to successfully achieve their individual academic goals of becoming lifelong learners through being supported in their academic visions, capacity building and professional advancement (Alshammari 2018). According to the TFL theory, authentic transformational leaders inspire their followers to be their better selves and pursue higher levels of self-achievement, thus achieving extraordinary results. They use inspirational motivation to foster commitment and loyalty in followers that is necessary in implementing transformation, which is vital in steering the organisation in new, compulsory directions while supporting them to reach optimal performance peaks (Jenkins 2021; Provitera & Sayyadi 2022).

Fifteen participants wished to achieve their own higher academic goals such as their master’s or doctoral degrees but were apprehensive because of the lack of management support. The wish, to some degree, requires one to ‘lead the self’ to easily acclimatise to the drastic changes that unnerve oneself (Cabatan et al. 2019). Self-leadership nurtures resilience, adaptability and productivity to higher heights through a sense of ownership and commitment. Furthermore, there are high levels of self-efficacy and proficiency all which direct nurse educators to advance their performance and achieve their academic goals (Kri et al. 2021). Nursing education institutions management must acknowledge that the research role of an academic is intimidating because of a lack of research confidence, primarily experienced by most nurse researcher neophytes without doctoral qualifications (Cabatan et al. 2019). Despite the intimidation, nurse educators are required to be research oriented and appropriately qualified, skills that present new challenging opportunities for nurse educators in this study. According to Bakker et al. (2022), Edrees et al. (2021) and Senekal and Lenz (2021), when new learning opportunities are created, authentic transformational leaders lend empowerment and individualised support where followers are treated as unique persons and their individual needs, abilities and differences are considered to assist them self-actualise. Therefore, their individual self-concept and self-worth are considered.

### Theme 3: Lack of support with minimal resources

All 20 nurse educators reported that there were inadequate human, material and technological resources at the NEI. The increased administration workload presented immense and numerous challenges for them because of an increase in student enrolment and a large number of repeaters. Instead of carrying out duties embedded in their multiple roles, nurse educators had to deal with other non-academic duties such as monitoring absenteeism and tracking sick notes from learners, scheduling classes, preparing students’ global reports, keeping archive reports and verifying results. Administrative work performed by nurse educators because of a shortage of NEI clerks resulted in job dissatisfaction as a result of increased workload and lack of focus in achieving and succeeding with the TLA activities (Coetzee 2019).

Nurse educators are expected to use innovative pedagogical strategies such as problem-based, authentic and peer-assisted learning that require technology to be used as a cognitive tool, a collaboration and communication enabler through which students can collaborate with anyone in the world, thereby become global citizens (Dube & Mlotshwa 2018). However, a shortage of technology personnel poses a challenge for both students and nurse educators to engage in TLA synchronously and asynchronously to develop students’ higher-order thinking skills. When nurse educators encounter technological challenges, they lose substantial time allocated for TLA activities, and thus, meaningful learning is not supported. Students cannot engage with information and communication technology specialists who could assist them in developing digital citizenship and the necessary research skills.

A lack of technological resources negatively affected TLA activities as nurse educators experienced difficulties in creating an environment of excellence, conducive to meaningful learning. Technology is an indispensable resource that has a significant and transformative impact in TLA activities (Shava 2022). Nurse educators were tasked with the responsibility to respond to technological changes effectively, efficiently and successfully by creating technology-rich learning environments that will provide for the academic needs of technologically savvy 21st-century graduates. Furthermore, they should manage the current resources and use strategies to acquire new appropriate resources to support NEIs’ transformational initiatives and improve TLA activities (Gupta & Gupta 2021). In accordance with the TFL style, authentic transformational leaders use *intellectual stimulation* to challenge their followers to bring creative, innovative solutions to solving organisational challenges. They are encouraged to think independently and outside the box about challenges while viewing long-standing problems from different perspectives (Bakker et al. 2022; Edrees et al. 2021).

### Strengths and limitations

The purpose of this research study was to gain a comprehensive understanding of management practices and their effect on TLA in a NEI in Gauteng. The qualitative, phenomenological research design utilised enabled the researcher to obtain insiders’ experiences, through exploring and describing the complex phenomenon of NEI management practises as reality lived and experienced by nurse educators. The research achieved its objectives and further highlighted important information with which good management practices at a NEI can be enhanced through recommendations. Therefore, this study added to the body of existing knowledge on effective management of NEIs with an aim to achieve individual and institutional goals within a transformative environment.

However, the majority of participants were female participants, and while the minimal male participation could be justified by their numbers in the current NEI, their experiences could have potentially brought in a distinct perspective about the effects of management practices on TLA activities. There was also a lack of diverse ethnic representation in the research study, and furthermore, the research was conducted with only nurse educators and not with NEI managers. Therefore, there was possibility for a biased sample attributable to the use of the purposive sampling method that could have enabled recruitment of only nurse educators reporting mostly negative experiences with the NEI management.

## Recommendations

### Nursing research

The researcher recommends that NEI managers are interviewed to obtain an unbiased global picture of challenges related to their practices within the NEI. A quantitative or mixed-method design could also be used to obtain a comprehensive sensible and corroborated understanding of nurse educators’ perceptions to mitigate the biases that can be associated with the qualitative method. Prospective researchers may purposively select more male nurse educators to explore and describe their experiences regarding the management practices and their effects on TLA activities.

### Nursing education and practice

Nursing education institution managers must be hands-on but flexible and may initially use the four ‘*I*’ s of TFL style to transform nurse educators but after success they may use a leadership style, which is suitable to the situation at hand particularly when working with a diverse group of nurse educators. Nursing education institution managers should be involved and constantly request nurse educators to evaluate their management practices through surveys to determine their strengths, weaknesses and areas of improvement. Communities of scholars must be created to provide support through sharing knowledge, networking, ideas, resources and coping strategies among nurse educators. Furthermore, they should foster a healthy work environment that reflects a high level of nurse educator participation in decision-making relating to pertinent issues that impact them to ensure that the future graduates are educated in a high-quality academic learning environment, and obligated transformation is a success in accordance with the global standards.

The NEI managers should ensure that workload and performance are monitored, and the teaching, administrative, research and community engagement duties are balanced. They should make rules and roles clear and apply them equally and fairly to all nurse educators to allay anxiety and perceptions of inconsistent treatment. A proper, mutual career plan and nurse educator academic needs analysis must be conducted, followed by an accountable staff development policy in line with the transformational changes in nursing education. Nursing education institution managers should look to establish a research collaboration and affiliation agreement between a NEI and a HEI to accelerate and facilitate professional and academic research development of nurse educators through research mentoring, engagement in community research projects, publications and the development of innovative pedagogical skills.

## Conclusion

This study revealed that nurse educators experienced management practices that were ineffective and had challenges in the current NEI. The ineffective practices of the management have a negative impact on the NEI in coping with major transformational changes brought by the complex, challenging and ever-changing nursing education environment. There are further challenges to develop a nursing graduate with necessary competencies to deal with the 21st-century challenges that include altruistic, individualised and comprehensive nursing care. Successful TLA activities are fundamentally driven by effective NEI management practices to attain success in achieving students’ and nurse educators’ lifelong academic goals. Knowing about these challenges, the findings of this research could assist NEI managers to enhance their management practices through adopting the four ‘*I*’ s of TFL, and thus, nurse educators’ TFL skills could develop. This, in turn, will make them appear as role models to their students, and thus, each individual’s and organisational goals can be achieved.
